# Dr. Storer's Case of Small-Pox after Inoculation

**Published:** 1811-11

**Authors:** John Storer

**Affiliations:** Nottingham


*To the Editors of the Medical and Physical Journal.*

GENTLEMEN,

A CASE of small-pox occurring in a boy who had, some years before, undergone *variolous* inoculation in a very perfect way, having been casually mentioned to me by Dr. Jaques, during a visit I made to Harrogate very lately, I inquired quired into the particulars, and finding them better authenticated than in any similar case that had come to my knowledge, I drew up a statement of it, solely with the view of its being inserted in the records of the public institution for extending the benefits of vaccination to the poor of this place, as an answer to objections sometimes urged against that practice, and usually founded on some instance of its failure, or supposed failure, of proving a perfect protection from subsequent small-pox.

The facts on both sides of this question, which in the true spirit of candour and observation have been so fairly stated in the last report of the national vaccine establishment, being well calculated to solicit the attention of the medical faculty and of the public to a dispassionate discussion of its merits, I conceive that the publication of the case alluded to, in your Journal, cannot fail to be useful and interesting at this moment. The facts narrated are supported by incontrovertible evidence, and are in their nature as conclusive as a single case can be supposed to furnish.

I am, Gentlemen,

Your obedient Servant,

JOHN STORER, M. D.

*Nottingham*, 10*th Sept.* 1811.

### CASE.

Thomas Lingforth, of Lower Harrogate, Yorkshire, 15 years of age, was inoculated in the spring of 1800, or 1801, by Mr. Wormdall, surgeon of Upper Harrogate, with variolous matter. The inoculated part made the usual progress; and at the accustomed period the variolous fever appeared, and was followed in a few days by a general eruption, which was considerable on the face, but accompanied with mild symptoms; and the whole disease disappeared in a favourable way, leaving a cicatrice at the part inoculated, which, on inspection, I found very perceptible at this day. The same boy being at the parochial school at Pannal, was sent on a message into the adjoining parish workhouse, where a child lay ill of the small-pox, and in a dying state. He was instantly sensible of a most offensive smell, of which he complained much, on his return to his father's house at Low Harrogate, on the same evening. This his father recollects to have happened in April, 1809, on a Tuesday; and on Friday of the same week, he was taken ill of head-ach, fever, &c.

Dr. Jaques, Physician at Harrogate, was desired to visit him; and being informed of his inoculation, and satisfactory progress under that process, eight or nine years before, he concluded from the nature and severity of the symptoms, that

 scarlatina, scarlatina, or some other eruptive disease, was impending.— On the following Monday an eruption appeared, so as to convince Dr. Jaques, who had been told of his exposure to the infection of small-pox, that it was truly variolous. This was confirmed beyond all controversy by the nature of the eruption, which proved confluent, and by the long continuance and severity of the disease from its commencement to its termination. His recovery in the end was perfect, leaving no bad consequences; and when it was to a certain degree advanced, three of his brothers and sisters, who had not before suffered this disease in any form, were taken with the small-pox, and all passed through it with more or less difficulty.

The above account I received separately from Dr. Jaques and Mr. Wormdall, the physician and surgeon who treated the patient at the times specified, from his father and mother; from his grandfather and grandmother, with whom he lived when he was inoculated; and from the young man himself, in respect to those particulars he was able to recollect. The marks of the casual small-pox are still apparent on his nose.

The facts are agreed upon by all the parties concerned, although the dates cannot be so accurately ascertained.

JOHN STORER.

